# Longevity-Extending MetAP2 Inhibitors Induce Caloric Restriction Through P53-Dependent Induction of GDF-15

**DOI:** 10.1093/geroni/igaa057.413

**Published:** 2020-12-16

**Authors:** Michael MacArthur, Sarah Mitchell, Jon Jung, Margaret Torrence, Alice Kane, Huseyin Mehmet, Brendan Manning, James Mitchell

**Affiliations:** 1 Harvard T.H. Chan School of Public Health, Boston, Massachusetts, United States; 2 University of Glasgow, Glasgow, Scotland, United Kingdom; 3 Harvard TH Chan School of Public Health, Boston, Massachusetts, United States; 4 Harvard Medical School Center for Animal Resources School of Public Health, Boston, Maine, United States; 5 Zafgen, Inc., Boston, Massachusetts, United States; 6 ETH Zurich, Zurich, Massachusetts, Switzerland

## Abstract

MetAP2 is a 67kDa protein which sits at the translation initiation complex and cleaves N-terminal methionine off of nascent peptides. Inhibitors of MetAP2 cause profound weight loss secondary to decreased food intake. These inhibitors also significantly extend longevity in mice in late-life intervention. However, the exact mechanism of action causing decreased food intake is not known. Here we investigated the molecular mechanism and target tissue of a MetAP2 inhibitor’s (Zgn1062) anorectic effects. First we identified the target tissue by testing targeted Zgn1062 delivery to specific brain regions. Delivery to the medio-basal hypothalamus did not have a significant effect but delivery to the lateral ventricle resulted in significantly decrease food intake and body weight after 2 and 14 hours. When we delivered a neuron-targeted AAV encoding MetAP2 shRNA we saw decreased efficacy of MetAP2 confirming the required for neuronal MetAP2 for anorectic effects. To determine the molecular mechanisms we performed RNAseq of wildtype and MetAP2 KO HT1080 cells across a timecourse of Zgn1062 treatment. The main pathway activated across timepoints in MetAP2-dependent manner was P53 signaling. A main P53 target that was upregulated was the known anorectic peptide GDF15. We confirmed GDF15 increases in vivo at both mRNA (liver and intestines) and protein level (serum) in response to Zgn1062. We also found that Zgn1062 treatment reduces senescent cell burden in visceral adipose tissue in vivo and reduces SASP gene expression in fat explants ex vivo. We hypothesize that Zgn1062’s potent P53 activation may play a role in clearance of senescent cells.

